# Hemodynamic Patterns of Spinal Cord Perfusion in Thoracoabdominal Aortic Aneurysm Repair

**DOI:** 10.1055/s-0041-1725121

**Published:** 2021-10-07

**Authors:** Giuseppe Rescigno, Carlo Banfi, Claudio Rossella, Stefano Nazari

**Affiliations:** 1 Foundation Alexis Carrel, Basiglio, Milan, Italy

**Keywords:** spinal cord perfusion, backflow, steal phenomenon, paraplegia, thoracoabdominal aortic repair, aneurysm

## Abstract

Paraplegia in aortic surgery is due to its impact on spinal cord perfusion whose hemodynamic patterns (SCPHP) are not clearly defined. Detailed morphological analysis of vascular network and collateral network modifications within Monro–Kellie postulate due to the fixed theca confines was performed to identify SCPHP. SCPHP may begin with intraspinal “backflow” (I-BF), that is, hemorrhage from anterior and posterior spinal arteries, backward via the connected anterior and posterior radicular medullary arteries, through the increasing diameter and decreasing resistance of segmental arteries (SAs), off their aortic orifices outside vascular network at 0 operative field pressure. The I-BF blood bypasses both intra- and extraspinal capillary networks and causes depressurization (0 diastolic pressure) and full ischemia of dependent spinal cord. When the occlusion of those SAs orifices arrests I-BF, the hemodynamic pattern of intraspinal “steal” (I-S) may take place. The formerly I-BF blood, in fact, is now variably shared between the fraction maintained in its physiological intraspinal network and that keeping flowing as I-S through the extraspinal capillary network. I-S is, however, counteracted by the extraspinal “steal” from the connected mammary/paraspinous-independent extraspinal feeders, all physically competing for the same room left by the missed physiological SA direct aortic blood inflow. Steal phenomenon evolves within the 120-hour time frame of CNm, whose intraspinal anatomical changes may offer the physical basis within the Monro–Kelly postulate, respectively of the intraoperative and postoperative paraplegia. The current procedures could not prevent the unphysiological SCPHP but awareness of details of their various features may offer the basis for improvements tailored, to the adopted intra- and postoperative procedures.

## Introduction

The extensive thoracoabdominal aortic aneurysm repair (TAAAR) carries a significant incidence of ischemic spinal cord injury, despite the evolution of surgical 1 and endovascular techniques. 2 After the introduction of the elephant trunk 3 technique, which may be viewed as the very first hybrid conceptual precursor of endovascular surgery, 4 endovascular techniques and newer hybrid techniques 5 were used virtually in all segments of the aorta.

Recent experimental studies showed significant modifications of the arterial intra- and extraspinal network in the immediate postoperative period, opening new perspectives 6 7 8 that may provide the basis for further speculative analysis of “the fascinating complexity of the intraspinal collateral flow pathways and might have modes of solid preoperative risk prediction of spinal ischemia in the future.” 6 a

## Pressure and Volume Changes in Blood and Spinal Fluid Compartments

**Fig. 1:**
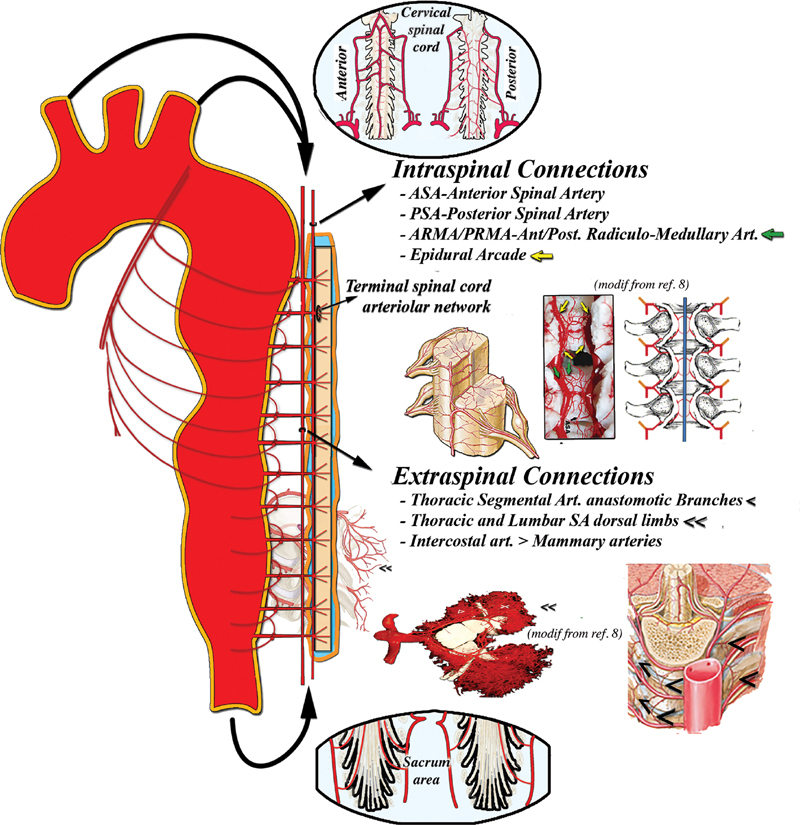
Spinal cord arterial networks. Schematic illustration of the spinal cord arterial supply system, relying on intraspinal and extraspinal networks longitudinally connected throughout the entire length of the spinal cord. Dissection layers, false channels, thrombi, embolism, etc., which are other factors potentially interfering with spinal cord perfusion hemodynamic pattern, were omitted in this and the following illustrations in the interest of focusing on the basic hemodynamic physiopathology. (This and all other figures contain elements modified from Netter Atlas of Human Anatomy, plates 168, Elsevier licensed NI0007432 September 9, 2016). SA, segmental artery.

The arterial blood supply to the spinal cord ( Fig. 1 ) comprises the thick intraspinal longitudinal network connecting terminal branches for the nervous tissue along the entire spinal cord mainly via the anterior spinal artery (ASA) and left and right posterolateral spinal arteries (PSAs).

An important although inconsistent and highly variable part of the arterial supply to the spinal cord is provided by the anterior radiculomedullary arteries (ARMAs, from 2 to 12 thoracic in humans 7 ), whose origins are part of the segmental artery (SA) trifurcation with two feeding branches to the circular epidural arcades, which are situated at the backside of each vertebral body, connecting the intraspinal arterial system longitudinally and side to side. 6 The posterior radiculomedullary arteries (PRMAs, from 11 to 16 in humans), 7 also supply the posterior part of the spinal cord.

At the thoracolumbar tract, an extraspinal network, formed by collateral prevertebral SA anastomotic branches and by a complex network mainly within the arterial branches of the paraspinous muscles, also longitudinally connects each spinal metamere.

Intercostal arteries themselves longitudinally connect the extraspinal network via the internal mammary arteries, as well as via anastomotic branches at the anterior surface of the vertebral bodies, providing a potentially direct aortic connection with the vascular supply of each spinal cord segment when SA are occluded.

Moreover, the central nervous system (CNS) has the peculiar feature of being entirely confined within a rigid osseous structure, the skull and vertebral canal, b 9 10 which necessarily implies a fixed total inner volume.

According to the Monro–Kellie postulate, the sum of the volumes of the brain–spinal cord tissue, cerebrospinal fluid, and intracranial–intraspinal blood is constant. 9 11 Being the CNS compartments in the physical liquid state (blood, cephalorachidian, and interstitial fluid) virtually incompressible, as it is generally assumed to be nearly so 11 the solid cell matrix of nervous tissue, the variation of the volume of any of CNS compartments necessarily implies the equivalent opposite variation of volumes of the others in or out the spinal canal, resulting in the constancy of their sum. 9 10 11 12 13 14 c

Although the relationship between the vascular supply to the spinal cord and the neurological complications associated with aortic surgery was thoroughly analyzed in the early years of aortic surgery, 15 the peculiar relationship within the CNS between the volume of spinal fluid and vascular perfusion in spinal cord injuries during thoracic aortic substitution was clinically acknowledged later. Clinical experience showed that patients could be protected against paraplegia and paraparesis 16 17 18 19 by draining enough spinal fluid preoperatively to keep its pressure ≤10 to 12 mm Hg throughout the operation and the postoperative period.

Moreover, several cases of paraplegia that appeared in the postoperative period fully recovered almost immediately following insertion of a spinal fluid drain, usually maintained for a few days and then removed. 20 21 The few milliliters of spinal fluid that were removed to bring the pressure to within 10 to 12 mm Hg implied a some way symmetrical increase mainly in blood volume within the spinal compartment that led, in these cases, to the recovery from paraplegia. These cases show then at least an approximate, indirect indication of the “extra” volume of the vascular network (capacity) of the spinal cord made available for blood flow after the spinal fluid was drained, the lack of which volume (capacity), wherever in the spinal canal having occurred, was shown to have been enough to cause paraplegia.

Hemodynamic patterns of spinal cord perfusion vary with the circulatory conditions that may be either physiological, modified or extracorporeal, and established during and after the repair that may differ in open or endovascular procedures, as well as by various surgical options, and may include periods of total circulatory arrest. All that may obviously variably impact on the hemodynamic models as outlined below, allowing however to accordingly predict the effects on them.

## The Backflow Phenomenon

The harmful effect of backflow from the aortic orifices of the SA in the tract between the clamps was first described, as “steal” phenomenon, by Wadouh et al 22 and the Hannover group in 1986. Experiments confirming the paraplegic effects of back bleeding, also still labeled as “steal,” were performed in a rabbit model by Kawanishi et al 23 at Kobe University. Back bleeding is occasionally considered to play a role, always indicated as “steal” phenomenon, in open procedures, 2 24 whereas its potential role in thoracic endovascular aneurysm repair (TEVAR), hybrid thoracoabdominal aortic repair (TAAAR), and conventional elephant trunk (cET) or frozen elephant trunk procedures has been ignored until recently. 25

## Backflow Pathways

**Fig. 2:**
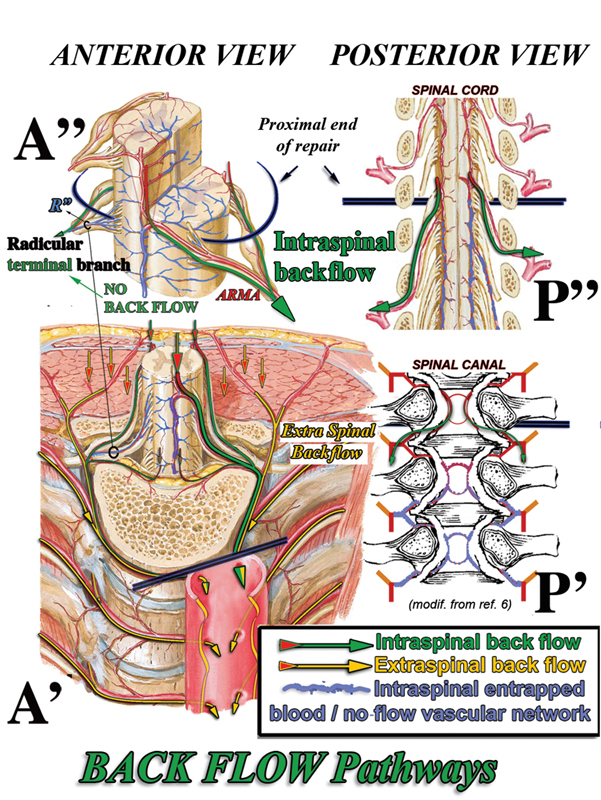
“Backflow” pathways. Intraspinal backflow can occur only through those segmental branches directly connected to the main longitudinal arterial axes (anterior spinal artery and posterior spinal artery) mainly via the anterior radiculomedullary artery (ARMA) and posterior radiculomedullary artery, as well as from any of the three segmental branches at each side of every metamere if not terminal to spinal tissue, including epidural arcades. The backflow blood bypasses both the intra- and extraspinal physiological capillary networks and being redirected toward the 0 (open procedures) pressure depressurizes the dependent spinal cord tissue where the blood is kept entrapped (Monro–Kellie postulate; see text and footnote ^e^ ). This and following figures illustrate the spinal cord perfusion hemodynamic pattern hypothesized mainly at proximal and possible distal ends of the substituted aorta. 6

Analysis of the arterial system excluded from direct SAs, aortic blood supply within the limits of aortic repair ( Fig. 2 ) permits one to predict that backflow from the aortic orifices of the SAs, which in case of hypothermic circulatory arrest may occur during resuming circulation, d comes mainly from the proximal (and possibly distal) confining integral parts (i.e., not included in the aortic tract under repair) of both the intra- and extraspinal arterial networks through their directly connected branches ( Fig. 2 ). 26

It is clear that arterial intraspinal “backflow” (I-BF) may occur only through those branches of the SA that are directly connected with the major longitudinal arterial axes of the spinal cord, that is, the ASA mainly via the connected ARMAs ( Fig. 2 , A' and A”) and left and right PSAs ( Fig. 2 , P”), mainly via the connected PRMAs. The tributary branches of the epidural arcades may also contribute to backflow 7 through the intraspinal longitudinal and side connections mainly at the proximal and possibly distal ends of the aortic tract under repair ( Fig. 2 , P').

Within the limits of the aortic tract under repair, the I-BF could not occur from all of the other arterial branches terminal to the spinal nervous tissue, including the terminal branches of the SA ( Fig. 2 [A”–R”]), terminal branches originating from the major longitudinal arterial axes, and those parts of the longitudinal axes that are no longer directly perfused ( Fig. 2 , arterial intraspinal network, outlined in blue); all of them in fact are bypassed by the proximal flow that is redirected toward the area of lower resistance and the 0 pressure open aortic orifices of the SAs.

In fact, the basic fluid mechanics underlying the Monro–Kellie postulate prevents the collapse of the intraspinal vascular compartment in conditions of no systemic pressure, thus keeping the blood entrapped in these terminal branches, the capillary network, as well as in the venous compartment 9 e ( Fig. 2 , paths outlined in blue).

Extraspinal “backflow” (E-BF) can be predicted to occur via the internal mammary system backward through the intercostal arteries to each SA aortic orifice along the aortic tract under repair, as well as backward via the paraspinous longitudinal and side-to-side arterial connections of the posterior branches of the SAs ( Fig. 2 , A', yellow paths and arrows).

## Backflow Phenomenon Physiopathology

Although backflow was first described as a steal phenomenon, 22 23 thus suggesting analogy with the usually benign subclavian steal syndrome that was well-known at time of its report, it actually implies a significantly different physiopathological pattern.

The subclavian steal syndrome in fact is characterized by the inverted direction of blood flow in the left vertebral artery toward the arterial network of the corresponding arm, due to the decreased or missed blood flow from the left subclavian artery caused by the stenosis/occlusion of its proximal tract. The resulting new hemodynamic equilibrium redirects the intrathecal “stolen” blood to the physiological capillary network of the left arm maintaining the systemic pressure in both segments and thus allowing some compensation by the peculiar physiological mechanisms of the CNS. 13

In contrast, in SA backflow, the back-flowing intrathecal arterial blood is redirected through arteries of increasing diameter and decreasing resistance toward the zero/− 2 H _2_ O cm negative intrathoracic pressure area outside the physiologically viable vascular system. That causes immediate depressurization (0 diastolic pressure) along the new backward pathway ( Fig. 2 , green paths) and then full bypass of the physiological capillary network of the dependent nervous tissue.

The comparison of the subclavian steal syndrome with SA backflow would imply that the vertebral artery, instead of simply inverting the direction of its blood flow, would be fully sectioned and its cranial stump left free to bleed outside the viable vascular system, which is exactly what occurs with SA backflow, then with the same full ischemic effects on the perfusion of the respectively dependent nervous tissues.

The backflow hemodynamic pattern then allows one to predict that most or all of the arterial blood supply via the longitudinal uninterrupted ASA could be lost through the first ARMA aortic orifice below the proximal interruption of the aorta, 22 ( Fig. 2 , A' and A”) thus causing the full bypass and depressurization of extended areas of the proximal and distal spinal cord as long as the backflow through the aortic orifices is allowed to freely occur. The same effects can be predicted to occur at the posterior spinal arteries via the PRMAs ( Fig. 2 , P”).

The backflow at aortic orifices with bypass of the respective physiological capillary network obviously includes the extraspinal network component that may continue to receive blood at each SA aortic open orifice backward via the internal thoracic artery and via the feeders of the paraspinous network, particularly at the ends of the aortic tract under repair ( Fig. 2 , A', yellow paths and arrows).

## The Steal Phenomenon Pathways and Physiopathology

**Fig. 3:**
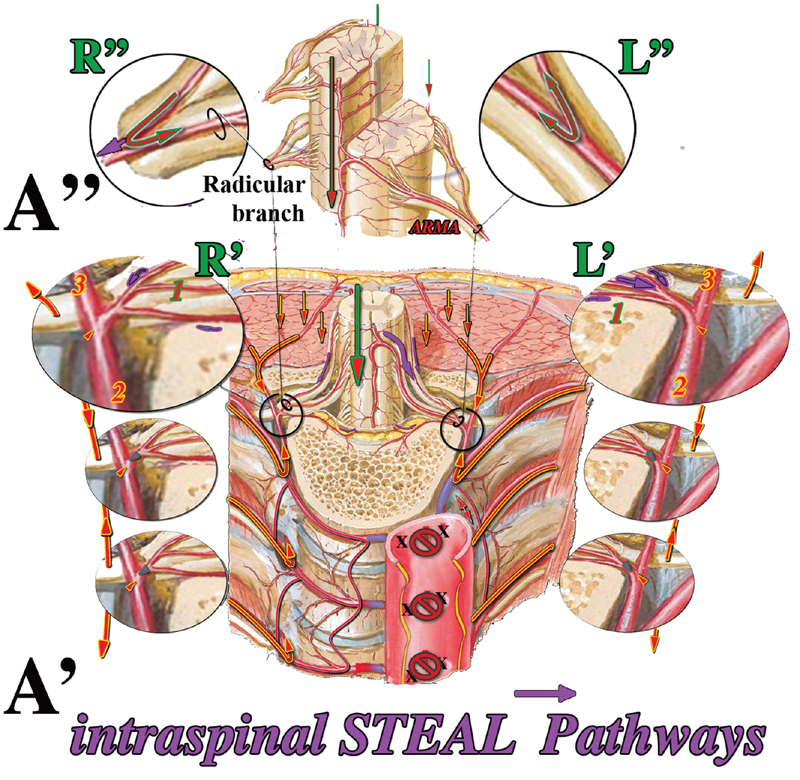
“Steal” pathways. When the aortic orifices are blocked, the missed direct aortic inflow at each spinal metamere all along the aortic tract under repair, leaves room for blood “stolen” to connected networks, that is, intraspinal network (1) and mammary/intercostal arteries (2) and paraspinal extraspinal (3) networks. As in vertebral steal, it is the inversion of the direction of flow, if any, at the common connecting branch between the intra and extra spinal arterial networks at each side of every spinal metamere the proof of the steal. ARMA, anterior radiculomedullary artery.

The opening of a new pathway of increasing diameter and decreasing resistance first created during aortic repair makes very clear the consequent direction of both the intra- and extraspinal arterial blood streams on both sides of all spinal metameres within the aortic tract under repair as well as the resulting full ischemia of the associated spinal cord segment(s). When the SAs aortic orifices are blocked instead, it is uncertain whether and how much, if any, of this formerly back-flowing blood that fully bypassed the physiological capillary network of both intra- and extraspinal networks can now be shared, at each side of every metamere, between the fractions that can be kept within its physiological intraspinal network ( Fig. 3 , A”, red arrows) and the fraction that remains to be redirected, as the steal phenomenon, toward the extraspinal capillary network ( Fig. 3 , A', R', and L', purple arrows) and possibly toward other branches of the intraspinal capillary network itself ( Fig. 3 , A”, R”, and L”).

Unlike the backflow, in the steal phenomenon, the “stolen” blood is not wasted but simply redirected toward a confining capillary network; diastolic pressure is preserved in both the intra- and extraspinal compartments, and then the potentially less-severe spinal cord perfusion impairment may be to some extent compensated. The therapeutically induced hypertension, which couldn't have any effect on spinal cord perfusion within the area bypassed by the backflow, can instead significantly improve or even restore the physiological spinal cord perfusion by a proportionally significant increase of blood flow in both the intra- and confining extraspinal physiological vascular networks.

The physical cause of the steal hemodynamic pattern is the missed aortic blood inflow from the SA which leaves proportional room at the extra spinal network for blood “stolen” to the confining, connected network feeders.

The more detailed analysis of the arterial network after the backflow is stopped shows ( Fig. 3 ) that at the unique and anatomically well-defined vascular branch that connects the intra- and extraspinal arterial networks at each side of every spinal metamere, three potential arterial pathways meet, each one fed by its own independent source ( Fig. 3 , A', circles L', and R' [1, 2, 3]) as follows: (1) spinal cord longitudinal axes via ARMAs and posterior communicating branches, (2) internal thoracic artery via the intercostal arteries, and (3) paraspinous arterial longitudinal anastomotic network 8 via the posterior branch of the SA.

The configuration of the resulting circulatory pattern is difficult or perhaps impossible to predict in practice. In theory, however, it is very clear that, at that well-defined common vascular branch at each side of every spinal metamere along the tract confining the aortic repair ( Fig. 3 , A', large black circles), the hemodynamic equilibrium between the intra- and extraspinal network flows must be settled somewhere between the two possible extremes, that is, from the worst clinical scenario of maximal intraspinal steal (I-S) to the reestablishing of the ideal physiological extra-to-intraspinal flow, in between being the no flow condition with occlusion due to the consequent thrombosis.

Whereas it could be interesting to know the respective blood flow and pressure of the mammary–intercostal arteries system ( Fig. 3 , nos. 2 in large ovals) and of the longitudinal paraspinous network source 6 7 8 ( Fig. 3 , nos. 3 in large ovals), the key point is then obviously the direction of blood flow, if any, at the common vascular branch at each side of every spinal metamere to or from the intraspinal vascular network, in particular at first or any ARMA within the aneurysmal aortic tract.

The intraspinal ischemic potential of the steal phenomenon eventually depends on the amount of blood stolen from the physiological intraspinal network that is added to the missed direct SA physiological inflow, which is in fact its direct physical cause.

Overall, it can then be speculated that the intraspinal “bad f steal” ( Fig. 3 , L') is counteracted by the extraspinal “good g steal” coming from the mammary–intercostal and from the paraspinous arterial feeders' networks competing for the same physical space made available for all three networks by the missed inflow from the SA orifices.

Hemodynamic patterns of spinal cord perfusion vary with the circulatory conditions that may be either physiological, modified, or extracorporeal, established during and after the repair that may differ in open or endovascular procedures, as well as by various surgical options, and may include periods of total circulatory arrest. All that may obviously and variably impact on the hemodynamic models as outlined above, allowing, however, to accordingly hypothesize the possible consequent effects on them.

## Collateral Network Modifications at 24 to 120 Hours

The backflow evolves into the steal phenomenon, eventually ending within the 120-hour time frame of the collateral network 8 27 28 29 30 that resumes reliable, if not necessarily physiological, spinal cord perfusion in at least 70% 29 of cases undergoing complete thoracoabdominal prosthetic substitution. Experimental studies on the collateral network showed important changes taking place soon at both the intra- and extraspinal vascular networks, confining the aortic repair, essentially characterized by the nearly doubling diameter of the ASA within 24 hours and of the epidural arcades, as well as the growth, ramification, and parallelization, of the paraspinal arterial network within 120 hours. The possible physiopathology nature of these changes, completed within different time frames, was discussed, and their possible different clinical effects were hypothesized, as immediate versus long-term spinal cord blood flow compensation, respectively. 7

**Fig. 4:**
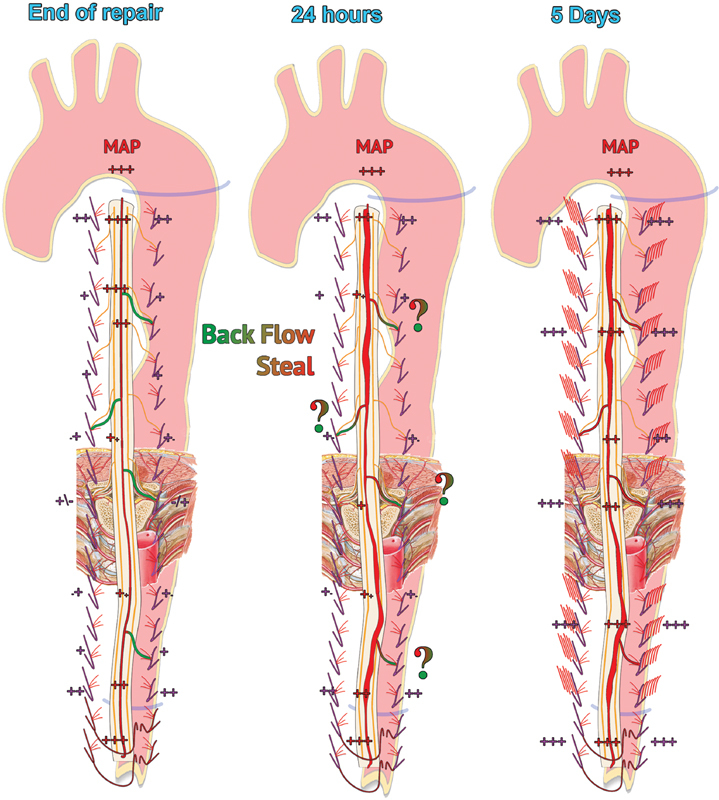
Sketches of collateral network (8) changes at 0 to 24 to 120 hours (morphology and pressure values are hypothetical). Beyond the related hypothetic spinal cord perfusion hemodynamic pattern these anatomical changes, very approximately schematically outlined here, may well fit, even in their respectively different time frames, with the symmetrically opposite changes in the pressure/volumes of blood and spinal fluid compartments physically implied by the fixed skeletal confines of the spinal cord (Monro–Kellie postulate) and could be a possible link to the onset of intraoperative (24-hour doubling anterior spinal art. diameter) and late postoperative (120-hour doubling epidural arcades diameter) paraplegia, respectively. MAP, mean arterial pressure; SA, segmental artery.

Whereas the actual impact of these anatomical changes ( Fig. 4 ) on the SCPHP may be difficult to quantify, the significant actual anatomical changes in the intraspinal arterial system (the doubling of the diameters of the ASA and epidural arcades) observed in experimental series may well fit, even in their respectively different time frames, with the necessarily, symmetrically opposite changes in the volumes of blood and spinal fluid within the respective compartments physically implied by the fixed skeletal confines of the spinal cord (Monro–Kellie postulate).

Accordingly one can hypothesize that perioperative spinal fluid drain may physically release critical cerebrospinal fluid pressure resulting from the doubling of the diameter of the ASA in the first 24 hours; on the other hand, the 120-hour time frame for the increase in the analogous diameter of the epidural arcades may well correlate with the onset of paraplegia postoperatively and the occasional recovery following prompt fluid drain. 20 21

## Discussion

**Fig. 5:**
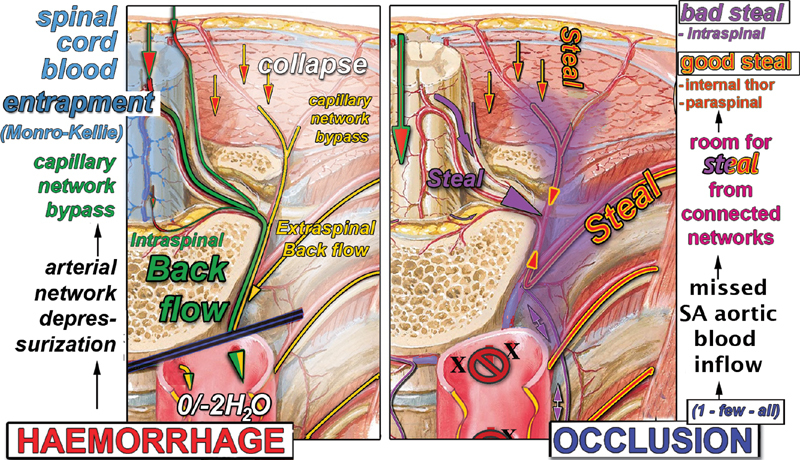
Hemorrhage and occlusion, the opposite causes of backflow and steal phenomena. Sketches of backflow and steal due to hemorrhage and occlusion of the segmental arteries (SAs) aortic orifices, respectively, occurring in sequential time frames, merging into one another during the first phases of the aortic repair. Whereas the “backflow” is always dangerously ischemic, the intraspinal bad “steal,” occurring to fill the room made available by the missed SAs aortic inflow, is counteracted by extra spinal “good” steal coming from mammary arteries and paraspinal networks. Moreover the blood entrapment into the spinal cord vascular network in the no-perfusion phase (Monroe–Kelly; see footnote ^e^ ) actually maintains the “priming volume” of the intraspinal arterial network throughout any ischemic phase, in contrast with the collapse occurring in any other parenchymatous organ when subject to ischemia. That results in the immediate tissue perfusion as soon as the circulation resumes that may perhaps play a role in the recovery from paraplegia after spinal fluid drain.

Backflow and steal phenomena are results of opposite events that may merge in phases of thoracoabdominal aortic repair ( Fig. 5 ).

Backflow, that is, the hemorrhage from a breach hemodynamically identical to the full section of a series of contiguous SA excluded from direct aortic perfusion, bypasses and depressurizes the connected networks and can end only by closure of the SA orifices or when no more space is physically available for the back-flowing blood. It occurs at the opening of the aorta and should be blocked as quickly as possible. Its very serious ischemic effects, however, can be open ended in thoracoabdominal surgical access by direct SA orifices oversewing and/or revascularization before irreversible lesions can occur.

Steal hemodynamic pattern is due to the obstruction of all or part of that series of contiguous SAs, whose missed direct aortic blood supply leaves room mostly at extra spinal network for retrieval of blood supplied by the independent feeders of the confining, much more extended, and connected intraspinal network, that is, “bad steal,” in competition with the “good steals” coming from the independent feeders of the extraspinal confining, connected internal thoracic and paraspinous arterial networks.

The complex anatomical configuration and the resulting variable hemodynamic patterns during and after repair do not yet allow to predict the eventual efficiency of spinal cord perfusion in the individual patient and, despite significant advancements, paraplegia can still occur with open or endovascular procedures.

The persisting clinical problem related to thoracoabdominal aortic repair contrasts with the situation in the brain during aortic arch repair, which was solved many years ago, despite the lower tolerance of the patient to ischemia of the brain than of the spinal cord. That may then suggest a comparison between these two vascular anatomical and hemodynamic patterns to try to hypothesize the underlying reasons for such different clinical outcomes.

In this ideal comparative hypothesis, in fact, compelling analogies can be easily identified between the vascular anatomical-hemodynamic configurations of the brain to that of each metamere of the spinal cord, whose anterior ischemia is enough to cause paraplegia.

The supraoptic trunks could represent in fact the analogous to the SAs at each spinal metamere, both providing extra- and intrathecal branches. The subclavian arteries could be viewed as the extrathecal branches analogue to the intercostal arteries at each side of every spinal segment, external carotids as the SA paraspinous posterior branches, and internal carotids being then the analogous of the common intrathecal branch of each side of every spinal metamere. Moreover, the internal carotid arteries join the circle of Willis, somehow as the intraspinal tract of SAs at each side of every metamere is posteriorly connected to the spinal epidural arcades, which were hypothesized 6 to be analogous to the circle of Willis to which are all longitudinally connected via vertebral art-ASA/PSAs axes.

**Fig. 6:**
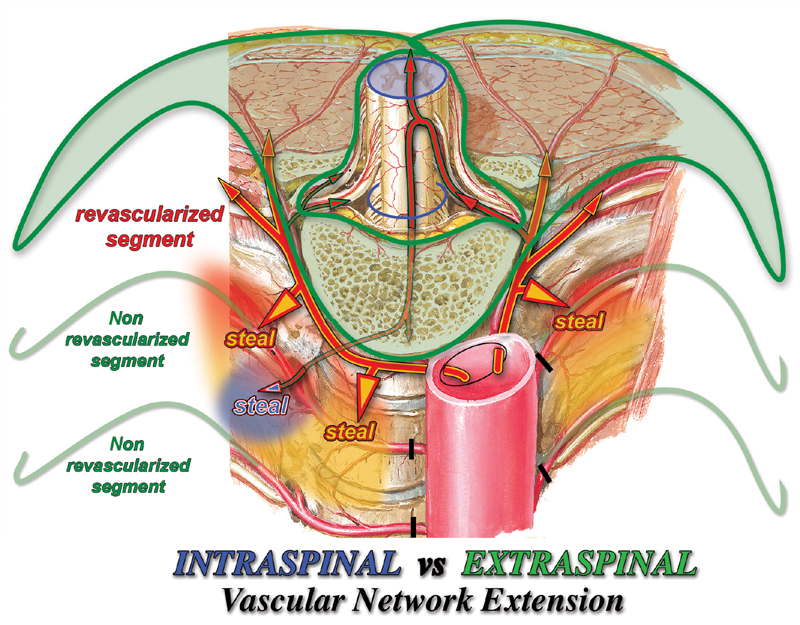
Intra- vs extraspinal arterial network extension: spinal cord perfusion hemodynamic pattern (SCPHP) of partial revascularization. Comparison of the respective extensions of the intra- and extraspinal vascular networks shows the prevalence in capacity of the extraspinal over the intraspinal network. It is then easily predictable that the blood supplied by a few revascularized segmental arteries (SAs) is directed predominantly to the extraspinal network of the confining nonrevascularized segments as steal phenomenon (yellow steal arrows) at the expenses of the spinal perfusion. Moreover even the blood actually entered the revascularized target anterior radiculomedullary artery (ARMA) may be eventually lost, as steal phenomenon (blue steal arrow), just at the next nonrevascularized ARMA segment, thus possibly explaining the inconstant clinical results of intraoperative partial revascularization or perfusion. SA revascularization adds anyway blood to the extraspinal network that, particularly if multiple, may directly or indirectly favorably impact and even reverse the above SCPHP.

These analogies of the vascular elements of the brain and spinal segment systems may provide an ideal model for more meaningful considerations of the two following points:

1. The multiconnected arterial networks of both the brain and each spinal segment can easily compensate for even multiple obstructions of nonterminal arterial branches. On the other hand, it is the multiconnection feature of the arterial networks itself that prevent the compensation for the extended depressurization of the hemorrhage (backflow) from even a small breach in the wall of any single extrathecal branch of either the arterial network of the brain or the spinal cord segment.
The ideal comparison of the common carotid arteries with the SAs of each spinal metamere may even better fit when considering the reperfusion phase where avoiding clots, debris and air embolism, and late false lumen thrombosis, all along the whole thoracoabdominal aortic tract in repair 31 h may then be as important as it is during carotid surgery. The spinal cord terminal branch occlusion caused by all these events in aortic repair, in fact and quite obviously, is out of the network potential compensation as that occuring during carotid surgery.
Moreover, reperfusion injuries 23 and/or any blood–spinal fluid Monro–Kellie volume conflicts are also obviously other possible, distinct paraplegia pathogenic events during aortic repair.
2. The comparison between the configurations of the brain and spinal cord arterial systems makes also clear the different proportional widths of their respective intra- and extrathecal compartments ( Fig. 6 ) and, more importantly, how that disproportion increases during partial revascularization (or temporary perfusion) in aortic repair only in the spinal cord.

In fact, the extrathecal arterial network of each spinal metamere ( Fig. 6 ) includes approximately the posterior half of the thoracic wall with the spine, whereas the corresponding intrathecal arterial network feeds the small spinal cord segment of most metameres, although may be somewhat extended on the side of the fewer metameres where ARMAs and PRMAs are present.

That difference favors the brain physiological intrathecal perfusion, when delivered through any of the current extracorporeal circulation techniques, also because its proportion with the extrathecal network does not vary during aortic arch repair.

On the contrary, when only selected SAs are revascularized or intraoperatively perfused, 24 the physiologically prevalent extrathecal extension compared with that intrathecal further extends into that of the confining, widely connected, and nonrevascularized spinal metameres. The resulting extraspinal steal (E-S; Fig. 6 , yellow steal arrows) by the confining nonrevascularized segments may then reduce or even nullify the intraspinal perfusion of the directly revascularized metamere. In fact, even the fraction of blood actually entering the target, revascularized ARMA may eventually be lost as steal just at the next nonrevascularized ARMA metamer ( Fig. 6 , blue steal arrow). On the other hand, the SA revascularization obviously adds volume to the extraspinal “good steal” that anyway may improve the eventual new SCPHP.

These considerations may allow us to predict that prevention of backflow requires appropriate maneuvers depending on the open operative technique used. The steal phenomenon, on the other hand, accordingly to its hemodynamic pattern cannot be fully prevented other than by reestablishing perfusion of all SAs ( Fig. 6 ).

Then full preservation of the whole SAs aortic perfusion with accurate prevention of any intraspinal thromboembolism is the ideal prerequisite to the paraplegia-free repair that is not yet constantly achieved with any of the current techniques and needs then shared efforts for applying new conceptual approaches. 32 33 34 35

## Conclusions

This article can be concluded with the following four points:

1. Backflow is defined as the free hemorrhage from the orifices of the SAs within the aortic tract under repair that causes depressurization (0 diastolic pressure), bypass of intra- and extraspinal arterial capillary networks, and then full ischemia of the dependent spinal cord tract. Backflow can occur in conjunction with any open extended aortic prosthetic repair procedure.
2. Steal phenomenon is caused by the occlusion of those same SAs, whose missed direct aortic blood supply leaves room at the extraspinal network for intraspinal blood “bad” steal at each side of every spinal metamere, particularly via critical ARMAs that is counteracted by extraspinal blood “good” steal potentially coming from mammary and paraspinal persistent feeders of extra spinal network. Steal phenomenon preserves diastolic pressure in both the intra- and extraspinal compartments and may be fully prevented only by keeping (cET) or restoring (revascularization) all 32 33 34 35 SAs direct aortic perfusion.
3. SAs anatomical analogies with common carotids may suggest that prevention of air, debris, clots, etc., embolism at each side of every spinal metamere is as important as in carotid surgery.
4. Reperfusion injuries and/or any blood–spinal fluid Monro–Kellie volumes conflicts are other possible, distinct paraplegia pathogenic events during aortic repair.

## Footnotes

a Speculative and prompted by contemporary author, to whom I also “gratia lubens refero (Gulielmo Harveo: Exercitaniones Duae Anatomicae De Circulatione Sanguinis ad Joannem Riolanum filium. Pag 1 line 12, Roterdami, Ex Officina Arnoldi Leers, 1649),” along a very old path not yet at its end!

## Footnotes

b The spinal cord has less rigid constraints on its total volume.

## Footnotes

c Alexandre Monro (1733–1817), with the Edinburgh group and others, proved this postulate in animal and human brain experiments hundreds of years ago. This elementary principle of the physical mechanics of fluids, as applied to the human CNS, underwent subsequent refinements, appraisals, and verifications from Monro (1783) to Cushing (1924) and was revisited with the introduction magnetic resonance. Since that time, many studies have been performed on the patterns and dynamics of these relationships in many diseases of the CNS, particularly hydrocephalus, to identify relevant conditions and normal values. A mathematical model was also developed to predict intracranial pressure gradients, blood and cerebrospinal flow patterns, and displacements in normal and pathological conditions, in particular, hydrocephalus. The effects of the systolic bolus of the blood compartment on the other compartments of the CNS have also been analyzed in detail. The autoregulation of the cerebral blood flow that occurs in response to changes in blood pressure was recently proved within ranges of pressure much narrower than traditionally (60–150 mm Hg) accepted.

## Footnotes

d During selective brain perfusion in hypothermic circulatory arrest however it may be hypothesized the backflow to occur at least at the proximal spinal cord.

## Footnotes

e Monro and Kellie, in fact, formulated their postulate from their original observation that in “experimental animals that had been bled to death only the brain does not appear pale or drained of blood.”

## Footnotes

f Unfavorable to the spinal cord perfusion, except the possible steals between intraspinal sectors that may not be necessarily “bad” for the spinal cord viability.

## Footnotes

g Favorable to the spinal cord perfusion.

## Footnotes

h That could be particularly relevant in hybrid arch-descending aorta repair, where it may offer an explanation for the unexpectedly baffling clinical results.
